# Soil-Transmitted Helminth Infection and Its Association with Anemia and Zinc Deficiency among Women in Nghe An Province, Vietnam

**Published:** 2019

**Authors:** Kieu-Anh THI TRAN, Ba-Loi CAO, Anh-Tram QUE, Tran-Anh LE

**Affiliations:** 1. Paediatric Department, Vinh Medical University, Vinh, Vietnam; 2. Scientific and Training Management Department, National Institute of Malariology, Parasitology and Entomology (NIMPE), Hanoi, Vietnam; 3. Department of Tropical disease, Vinh Hospital of Friendship General, Vinh, Vietnam; 4. Department of Parasitology, Vietnam Military Medical University, Hanoi, Vietnam

## Dear Editor-in-Chief

Soil-transmitted helminth (STH) infections are prevalent worldwide, especially in tropical regions such as sub-Saharan Africa, the Americas, China and East Asia (1). Among STH, the most common worms are *Ascaris lumbricoides* (roundworm), *Trichuris trichiura* (whipworm) and hookworms (*Necator americanus* and *Ancylostoma duodenale*). STH infection is related to anaemia or deficiency of some micronutrients such as zinc (Zn), selenium (2). The severity of nutritional effect depends on the intensity of infection and the host's status. Women of childbearing age and children are among the most vulnerable groups since they have a high demand for nutrients (3). The strategy for control of STH infections is to control morbidity through the periodic treatment of at-risk people living in endemic areas so the knowledge of epidemiological characteristics of STH infection as well as anaemia in different regions is needed to build the appropriate strategy for infection control (1). Vietnam is a developing country located in Southeast Asia, a region with many environmental and socioeconomic factors that promote the infection of STH in the human population.

Nghe An is a central province of Vietnam where there are many factors favourable for the transmission of STH but the most recent report was in 2006 with the prevalence of STH infection was 81% (4). This raises the need to investigate the prevalence and impact of STH infection on nutritional status among women of childbearing age, considered at-risk population, in Nghe An Province, Vietnam.

This cross-sectional study was carried out between Jan 2014 and Dec 2016 in Dien Chau district, Nghe An Province, Vietnam. Overall, 216 pregnant (mean age of gestation was 5.33 wk) and 204 non-pregnant, expecting-child women were involved in the study.

Informed consent was taken from the cases and the study was approved by local Ethics Committee.

Helminth parasite examination was performed using Kato-Katz thick smear methods. Haemoglobin (Hb) concentration was estimated with Hemocue microcuvettetes (Hb 201 kit) and serum Zn was assessed by atomic absorption spectrophotometry. Nearly half (49.52%) of participants were infected with at least one species of pathogenic intestinal helminths. The prevalence of *N. americanus/A. duodenale* infection was highest (30.95%) and followed by *T. trichiura* (18.1%), *A. lumbricoides* (6.19%) and most of the infected women were categorized as light infection. The difference between the prevalence of STH in pregnant and non-pregnant women was not statistically significant. The rates of anaemia and Zn deficiency among participants were 8.81% and 29.05% respectively. Pregnant women had a lower concentration of Hb and Zn compared to that of non-pregnant women (12.67±1.33 mg/dL and 11.22 ±2.13 μg/L vs. 13.51±1.32 mg/dL and 12.67±2.49 μg/L, respectively). There was no difference between the rates of anaemia (8.33 vs. 9.31%; *P*=0.86) but the rate of Zn deficiency among pregnant women was significantly higher than that among non-pregnant women (39.45% vs. 18.14%, *P*<0.001). No association was observed between anaemia and the rate of Zn deficiency with any species of helminth among participants. The lower level of Hb and Zn in pregnant women caused by pregnancy but not by helminth infection. The reason was possibly due to the fact that almost all infected women classified as light infection because the impact of STH infection on nutritional status is related to worm burden and light infections may have no relation with anaemia but moderate or heavy infection can cause anaemia (5). Lower Hb concentration during pregnancy was due to the increase of the total blood plasma volume so that the number of erythrocytes per unit of blood and Hb level decline (6). Among pregnant women, there have been some reports showing a negative association between HI and Zn level (3). However, in the current study, we could not find any association between STH infection and Zn deficiency but the rate of Zn deficiency was found to be nearly 2-fold higher in pregnant women than in non-pregnant women (*P*<0.001). Zn deficiency among local women was related to pregnancy which was in line with results of some other studies (7, 8). The most significant determinants for the development of Zn deficiency were inadequate intake of Zn and elevated Zn requirements (9). The population at higher risk of Zn deficiency are those with increased requirements for Zn such as infants, children, adolescents, pregnant and lactating women but not those with helminth infection (10).

The findings of this study demonstrated the high prevalence of light-intensity STH infection that not related to anaemia and Zn deficiency among women living in a central province of Vietnam. These findings can be used to design health plans for women in Nghe An or other regions with similar epidemiological characteristics which focus on providing iron and Zn supplement irrespective of helminth infection.
